# A deep learning approach to dysphagia-aspiration detecting algorithm through pre- and post-swallowing voice changes

**DOI:** 10.3389/fbioe.2024.1433087

**Published:** 2024-08-02

**Authors:** Jung-Min Kim, Min-Seop Kim, Sun-Young Choi, Kyogu Lee, Ju Seok Ryu

**Affiliations:** ^1^ Department of Health Science and Technology, Graduate School of Convergence Science and Technology, Seoul National University, Seoul, Republic of Korea; ^2^ Department of Rehabilitation Medicine, Seoul National University Bundang Hospital, Seongnam, Republic of Korea; ^3^ Department of Multimedia Engineering, Dongguk University, Seoul, Republic of Korea; ^4^ Music and Audio Research Group, Department of Intelligence and Information, Graduate School of Convergence Science and Technology, Seoul National University, Seoul, Republic of Korea; ^5^ Seoul National University College of Medicine, Seoul, Republic of Korea

**Keywords:** dysphagia-aspiration, aspiration detection model, voice changes pre-and post-swallowing, deep learning, voice-based non-face-to-face monitoring

## Abstract

**Introduction:**

This study aimed to identify differences in voice characteristics and changes between patients with dysphagia-aspiration and healthy individuals using a deep learning model, with a focus on under-researched areas of pre- and post-swallowing voice changes in patients with dysphagia. We hypothesized that these variations may be due to weakened muscles and blocked airways in patients with dysphagia.

**Methods:**

A prospective cohort study was conducted on 198 participants aged >40 years at the Seoul National University Bundang Hospital from October 2021 to February 2023. Pre- and post-swallowing voice data of the participants were converted to a 64-kbps mp3 format, and all voice data were trimmed to a length of 2 s. The data were divided for 10-fold cross-validation and stored in HDF5 format with anonymized IDs and labels for the normal and aspiration groups. During preprocessing, the data were converted to Mel spectrograms, and the EfficientAT model was modified using the final layer of MobileNetV3 to effectively detect voice changes and analyze pre- and post-swallowing voices. This enabled the model to probabilistically categorize new patient voices as normal or aspirated.

**Results:**

In a study of the machine-learning model for aspiration detection, area under the receiver operating characteristic curve (AUC) values were analyzed across sexes under different configurations. The average AUC values for males ranged from 0.8117 to 0.8319, with the best performance achieved at a learning rate of 3.00e-5 and a batch size of 16. The average AUC values for females improved from 0.6975 to 0.7331, with the best performance observed at a learning rate of 5.00e-5 and a batch size of 32. As there were fewer female participants, a combined model was developed to maintain the sex balance. In the combined model, the average AUC values ranged from 0.7746 to 0.7997, and optimal performance was achieved at a learning rate of 3.00e-5 and a batch size of 16.

**Conclusion:**

This study evaluated a voice analysis-based program to detect pre- and post-swallowing changes in patients with dysphagia, potentially aiding in real-time monitoring. Such a system can provide healthcare professionals with daily insights into the conditions of patients, allowing for personalized interventions.

**Clinical Trial Registration:**

ClinicalTrials.gov, identifier NCT05149976

## 1 Introduction

Dysphagia refers to a spectrum of abnormalities that occur during the entire swallowing process, including the oral, pharyngeal, and esophageal stages (Matsuo and Palmer, 2008; Saitoh et al., 2018). Videofluoroscopic swallowing study (VFSS) is regarded as the most standardized diagnostic method for assessing dysphagia (Tohara et al., 2003; Clave et al., 2004; O'Horo et al., 2015). Medical professionals utilize standardized protocols based on VFSS images, such as the Penetration-Aspiration Scale (PAS) (Rosenbek et al., 1996; Robbins et al., 1999; Borders and Brates, 2020), which is a widely accepted scale in clinical practice for evaluating the presence of residues around the larynx and the occurrence of aspiration by assigning grades based on the severity of food penetration or aspiration into the airway or vocal cords (Rosenbek et al., 1996; Robbins et al., 1999). Aside from VFSS, other methods for diagnosing dysphagia include fiberoptic endoscopic evaluation of swallowing, high-resolution manometry, and tongue pressure measurement (Lind, 2003; Martin-Harris and Jones, 2008; Vaiman and Eviatar, 2009; Abdel Jalil et al., 2015; Jayatilake et al., 2015; Reynolds et al., 2016; Langmore, 2017; Saitoh et al., 2018; O'Brien et al., 2021; Helliwell et al., 2023). However, these diagnostic methods require patients to visit medical facilities with the necessary equipment, carry the risk of radiation exposure, and have limitations in periodically monitoring the constantly changing condition of patients with swallowing disorders (Martin-Harris and Jones, 2008; Vaiman and Eviatar, 2009; Jayatilake et al., 2015; Reynolds et al., 2016; Langmore, 2017; Saitoh et al., 2018; O'Brien et al., 2021; Helliwell et al., 2023).

To overcome these limitations, several studies have been conducted to detect dysphagia using patients’ voices. Several studies have explored wet phonation as a risk factor for penetration and aspiration (Warms and Richards, 2000; Groves-Wright et al., 2010; Santos et al., 2015). Additionally, previous reports have investigated whether voice indicators, including frequency and amplitude variability, noise-to-harmonics ratio, voice intensity, and duration, change pre- and post-swallowing substances by comparing patients with dysphagia, particularly those experiencing aspiration, with healthy individuals and have suggested that food accumulation affects vocal cords vibrations and voice quality, potentially altering the voice patterns (Ryu et al., 2004; Waito et al., 2011; Kang et al., 2018; Dos Santos et al., 2022; Song et al., 2022). Nonetheless, there remains a paucity of studies assessing pre- and post-swallowing changes owing to the need for researchers to analyze voice indicators using speech analysis software, which has limitations in developing medical devices for monitoring patients’ daily lives in clinical settings.

Based on previous research on the relationship between dysphagia and voice analysis, our research team previously developed an algorithm for detecting aspiration in dysphagia using only post-swallowing voice data. This algorithm was developed using the MobileNetV3-based Efficient Pre-trained CNNs for Audio Pattern Recognition (EfficientAT model, MIT license). Using the best-performing mn30_as model (mn: MobileNetV3, 30: width multiplier, mn30_as: pre-trained model), it achieved an average AUC of 0.8010 for the male model, 0.7572 for the female model, and 0.8361 for the combined male and female model. (Schmid et al., 2023a; 2023b; Kim et al., 2024). However, the previous study had several limitations. First, it could not detect changes in voice before and after swallowing, limiting the fundamental intervention and diagnosis of dysphagia. Second, using only post-swallowing meals voice data restricted the number of data samples, limiting the model’s generalization ability. There were also issues with the research protocol. The 5-s ‘ah∼’ vocalization was challenging for elderly dysphagia patients, leading to reduced standardization of voice data length. Additionally, noise removal between voice segments was incomplete.

In this study, we aim to overcome these limitations. We utilized both pre- and post-swallowing voice data to detect changes, standardized voice data into 2-s units to improve data quality, amplified learning data through combinations of pre- and post-swallowing voices, and applied more sophisticated noise removal methods. These improvements were expected to enhance the accuracy of aspiration detection in dysphagia and improve the model’s generalization ability. Clinically, detecting changes pre- and post-swallowing will contribute more effectively to the early diagnosis and intervention of dysphagia. This represents a significant advancement in overcoming the limitations of existing research and advancing the technology of dysphagia detection through voice analysis.

The hypotheses of this study were (i) that variations in patients’ voices pre- and post-swallowing might be indicative of the presence or absence of aspiration in the pharynx or larynx after eating and (ii) that these patterns would differ from those in healthy individuals. With these hypotheses in mind, the current study primarily aimed to construct a machine-learning algorithm capable of detecting voice alterations pre- and post-swallowing, which would enable differentiation from healthy individuals and facilitate real-world patient monitoring.

## 2 Materials and methods

### 2.1 Study design

This prospective cohort study was conducted from October 2021 to February 2023 at the Seoul National University Bundang Hospital. This study was approved by the Seoul National University Bundang Hospital Institutional Review Board (protocol ID: B-2109-707-303, first approval date: 2021.09.01, actual study start date: 2021.10.07) and adhered to the Strengthening the Reporting of Observational Studies in Epidemiology (STROBE) guidelines. All participants received a thorough explanation about the study and subsequently provided informed consent. The study was registered at ClinicalTrials.gov (ID: NCT05149976) and strictly followed the approved research protocols and guidelines.

### 2.2 Participants

The study participants were selected from patients who were scheduled to undergo VFSS at our hospital for symptoms and signs of dysphagia and from healthy participants who were recruited through hospital announcements and various media outlets. The inclusion criteria for this study were as follows: (i) patients scheduled to undergo VFSS, (ii) patients capable of recording their voice while saying “ah∼” for 5 s, and (iii) healthy participants (those without signs of dysphagia) capable of voice recording. The exclusion criteria were as follows: (i) patients who were unable to phonate; (ii) patients who underwent VFSS re-examination; (iii) patients with voice-related disorders (e.g., dysphonia, polyps, vocal cord paralysis); (iv) participants who did not record their voice both pre- and post-swallowing; (v) participants deemed unsuitable for the study by the researchers; (vi) participants who had recordings with background noise or other people’s voices louder than the participants; and (vii) participants with poor-quality recordings. Only those who agreed to participate in the study after seeing the recruitment notice were chosen as the study participants. The suitability of all study participants was determined through a survey conducted by three healthcare professionals (a clinical dietitian, an occupational therapist, and a clinical physician) specializing in dysphagia. For patients who underwent VFSS, interpretation was performed by two clinical physicians. The reliability of the interpretations of these two experts was determined using Cohen’s kappa coefficient, which indicated a value of 0.87. Selection was based on factors such as age, underlying medical conditions, sex, dysphagia-related symptoms, and VFSS findings. The final eligibility of participants was determined based on the judgment of two clinical physicians.

**FIGURE 1:**
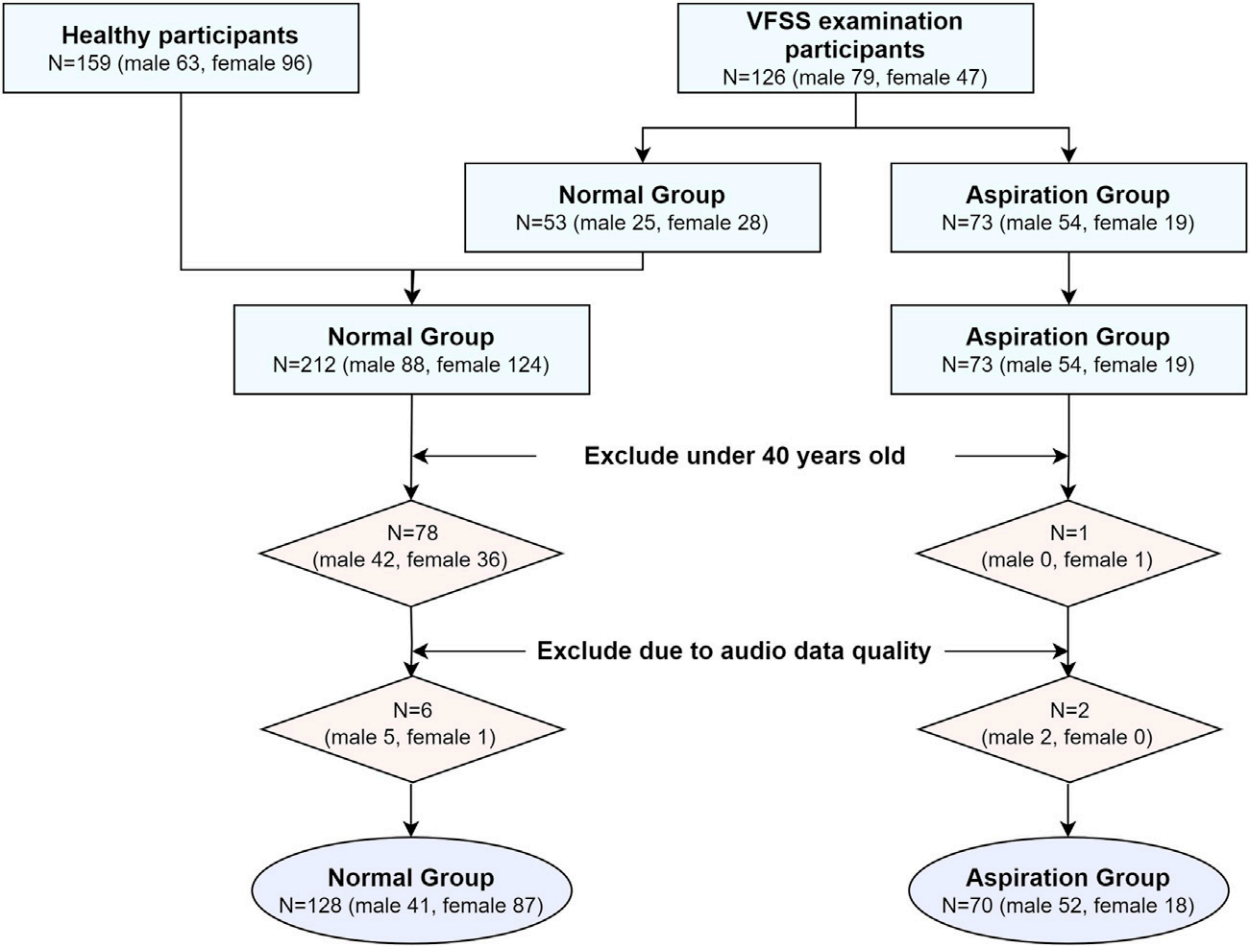
Study flow for the participant selection.

A total of 159 participants without dysphagia symptoms and 126 participants who underwent VFSS for such symptoms were included in this study. The voices of these 285 participants were recorded pre- and post-swallowing. Among these participants, 159 healthy participants and 53 VFSS examinees were assigned to the normal group (PAS score of 1), whereas 73 participants were classified as the aspiration group (PAS score of 5–7). Participants below 40 years of age were excluded to avoid age-related bias, resulting in the exclusion of 78 participants from the normal group and one participant from the aspiration group. Consequently, the final cohort included 134 and 72 participants in the normal and aspiration groups, respectively. However, owing to audio quality issues, six more participants in the normal group and two participants in the aspiration group were excluded, leaving 128 and 70 participants in the normal and aspiration groups, respectively, for the final analysis. Figure 1 presents the study flow of the participants in this study.

### 2.3 Data collection

After screening for eligibility and obtaining informed consent, the participants vocalized “ah∼” for 5 s pre- and post-swallowing various substances. The VFSS examinees consumed water, yoplait yogurt (YP), small fluid (SF), semi-blended diet (SBD), fluid thickening with level 3 (FT3), and liquid food (LF), whereas the healthy participants only consumed water. For the VFSS examinees, recordings were performed in stereo using a Sony ICD-TX660 recorder at a 16-bit depth and bit rate of 64 kbps, with sampling at 44.1 kHz and with a microphone capturing a frequency range of 95–20,000 Hz. For the healthy participants, recordings were carried out using personal mobile phones. The intake of each substance was limited to 3 cc. The researchers provided instructions to the VFSS examinees through an intercom system in the VFSS room to maintain a noise-free environment, with the recording device placed on the participants’ sleeves. Recordings for the healthy participants were also performed in an isolated room with minimal noise under supervision.

Recordings for the same individual were conducted using different devices (Samsung mobile phone, iPhone, and Sony voice recorder) in the same environment to minimize recording device-related bias between the healthy participants and the VFSS examinees. Furthermore, the impact of device bias was determined by measuring the cosine similarity after applying the preprocessing steps, including the transformation of data into Mel spectrograms. The results showed similarity scores of 0.9491 for the Samsung mobile phone *versus* iPhone, 0.8683 for the Samsung mobile phone *versus* Sony recorder, and 0.9531 for the iPhone *versus* Sony recorder. Despite the challenges in recruiting a hospital-based normal control group, bias was diligently addressed through preprocessing to ensure data integrity. The normal group recorded 133 files (pre: 69, post: 64) for males and 299 files (pre: 153, post: 146) for females, whereas the aspiration group contributed 242 files (pre: 95, post: 147) for males and 79 files (pre: 33, post: 46) for females.

### 2.4 Voice data cleaning and transformation

In our study, the patient voice data underwent a six-stage transformation for machine learning: (1) The data of all participants were initially denoised by removing any external sounds, such as voices or equipment noises found before and after the 5-s recording, by trimming the start and end of the recordings. (2) The voice recording protocol set the recording time to 5 s; however, there were instances in which the recordings were shorter or longer than 5 s, depending on the condition of the participants. For standardization and augmentation, all data were trimmed to 2-s intervals. This process effectively removed any noise that was present within the 5-s recording period. (3) The voice files were recorded in various formats, such as mp3, m4a, and wav. With the future development of medical devices and mobile platforms in mind, all files were converted to the 64-kbps mp3 and mono format to standardize the data format. The stereo files from the Sony recorder were split into left and right channels and converted to mono to facilitate model training, ensuring compatibility with machine learning algorithms and standardizing recording formats across different environments. After this conversion, the normal group had 235 pre-recordings and 240 post-recordings for males and 531 pre-recordings and 553 post-recordings for females. The aspiration group had 266 pre-recordings and 444 post-recordings for males and 96 pre-recordings and 134 post-recordings for females. (4) Based on the voice data obtained from the same individual simultaneously pre- and post-swallowing, combinations were created, resulting in 1,556 pairings for males in the normal group, 3,777 pairings for females in the normal group, 2,200 pairings for males in the aspiration group, and 768 pairings for females in the aspiration group. (5) Subsequently, the dataset was divided into ten random segments based on individuals (anonymized identifiers) and structured into training and test sets in hierarchical data format 5 (HDF5) format containing anonymized identifiers, labels, and pre- and post-audio data. Bias was further mitigated by placing the voice samples from the same individual (anonymized identifiers) in the same fold. Due to group disparities among the participants and limitations in sample size, a 10-fold cross-validation was employed. (6) A significant imbalance in data combinations for females was observed between the normal and aspiration groups. In order to address this, random oversampling based on normal data was performed for each fold, but only for the training dataset of the female models. Additionally, in the combined model, oversampling was applied exclusively to the female data.

### 2.5 Voice data preprocessing

**FIGURE 2:**
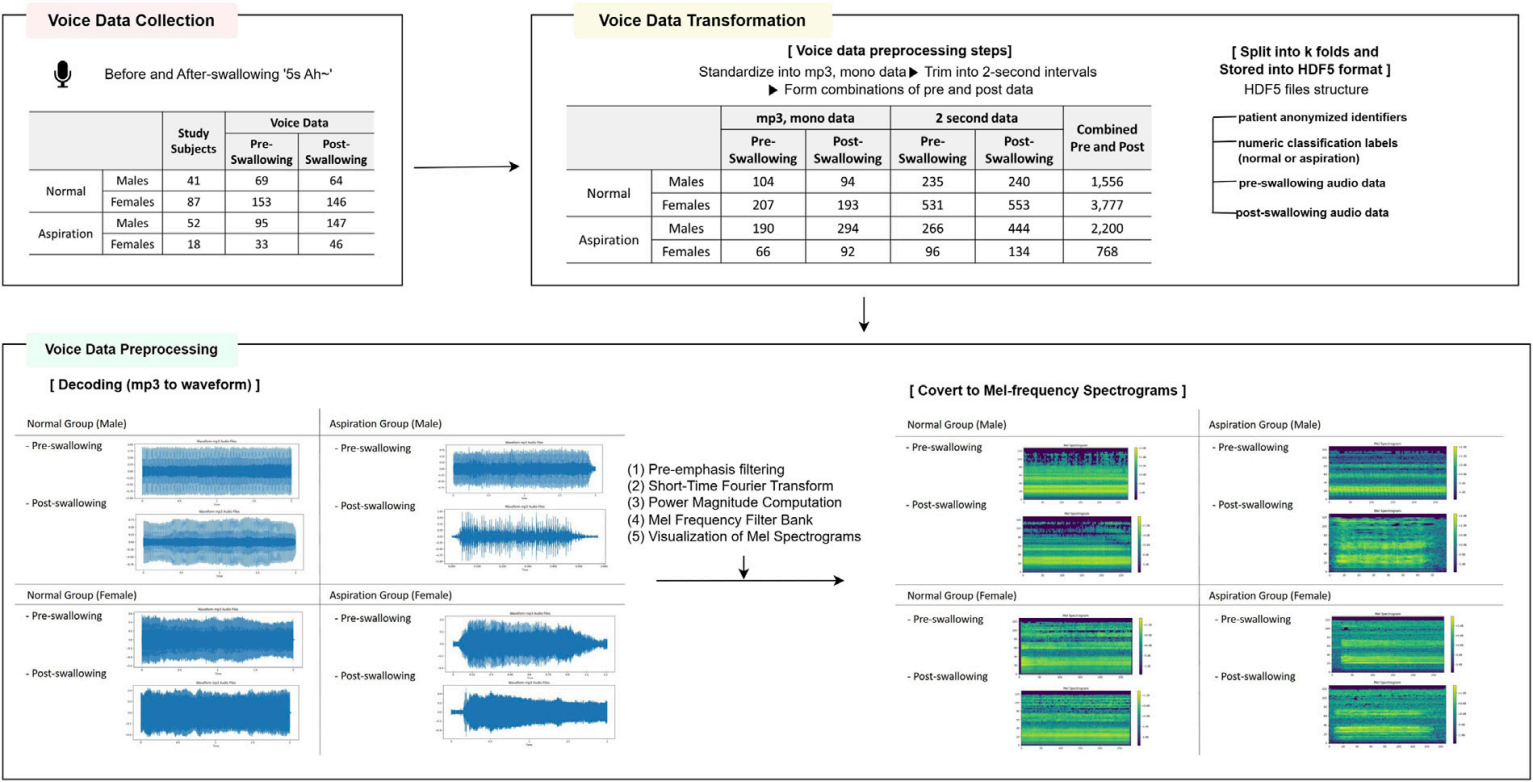
Voice data transformation and preprocessing.

Before training the machine-learning model with patient voice data, preprocessing was performed based on the code from the Efficient Pre-trained CNNs for Audio Pattern Recognition (EfficientAT model, MIT license) (Schmid et al., 2023a; 2023b). This process was implemented in PyTorch and transformed the voice data into a visual feature format, specifically Mel spectrograms, to be used as input data for the machine-learning model. The integrity of the recorded audio data was ensured by undertaking several measures to minimize noise interference. Recordings were performed in a controlled, soundproof environment and were manually reviewed to eliminate sections with substantial background noise or mechanical disturbances that could potentially hinder subsequent analysis. Additionally, a series of preprocessing steps was applied to convert the recorded audio from the waveform to Mel spectrogram format; these steps included pre-emphasis filtering, short-time Fourier transform, power magnitude computation, and Mel frequency filter bank processing. The default input parameters for this process were set as follows: number of mels, 128; sampling rate, 32,000; window length, 640 (20 m); hop size, 320 (10 m), and number of fast Fourier transforms (640). Figure 2 shows the voice data collection, transformation, and preprocessing process in this study.

### 2.6 Development of the voice-change detection model

The preprocessed data were trained using a modified version of the EfficientAT model (Schmid et al., 2023a; 2023b). The original EfficientAT model was optimized for classification tasks on audio data. However, our research model was designed to detect not only normal and aspirated voices but also changes pre- and post-swallowing. Our modifications to the MobileNetV3 architecture focused on voice change detection, including (1) sequential data handling for separate feature extraction from pre- and post-swallowing audio data, (2) encoding for efficient audio feature extraction from both states, (3) channel separation to process audio data individually, (4) tensor transformation to create distinct layers for each audio type, (5) a decoder for improved voice change detection using the last convolution layers and specialized blocks, (6) significant channel expansion (first 6x, then 2x, resulting in a total 12x expansion) to increase model expressiveness, (7) feature concatenation integrate information from both pre- and post-swallowing states, and (8) enhanced training with Mel spectrogram-formatted data. This adaptation created a versatile MobileNetV3 implementation that emphasized voice change detection and included a fully convolutional head type, BatchNorm2d layers to normalization, 12 workers for data processing, 150 training epochs, and a model width of 2.0, aligning with the “width multiplier” parameter of EfficientAT (Schmid et al., 2023a; 2023b). To improve the generalization performance on the training dataset, dynamic audio sample augmentation was applied by randomly selecting two out of the following seven data augmentation methods: (1) adding the Gaussian noise, (2) adding the Gaussian noise to adjust the signal-to-noise ratio, (3) adjusting the audio volume, (4) inverting the polarity of the audio signal, (5) distorting the audio signal using the hyperbolic tangent function, (6) masking certain time intervals, and (7) stretching or compressing the playback time to introduce temporal distortion to the audio samples.

The learning rate was controlled using a ‘LamdaLR’ learning-rate scheduler. It started at the specified learning rate (initial learning rate: 5.00e-5 or 3.00e-5) with the Adam optimizer, remained constant initially, and then began to decrease linearly from epochs 100 to 105, ultimately reaching the final learning rate of the initial learning rate multiplied by 0.01. A batch size of either 16 or 32 was used. For the loss function, we employed binary cross-entropy with logits. This choice is particularly suitable for our binary classification task, providing both numerical stability and computational efficiency. Despite attempting L2 regularization due to limited dataset size, the best performance was achieved without regularization, likely due to constraints imposed by dataset size. Therefore, regularization was not applied in this study.

### 2.7 Inference: detection of dysphagia-aspiration

**FIGURE 3:**
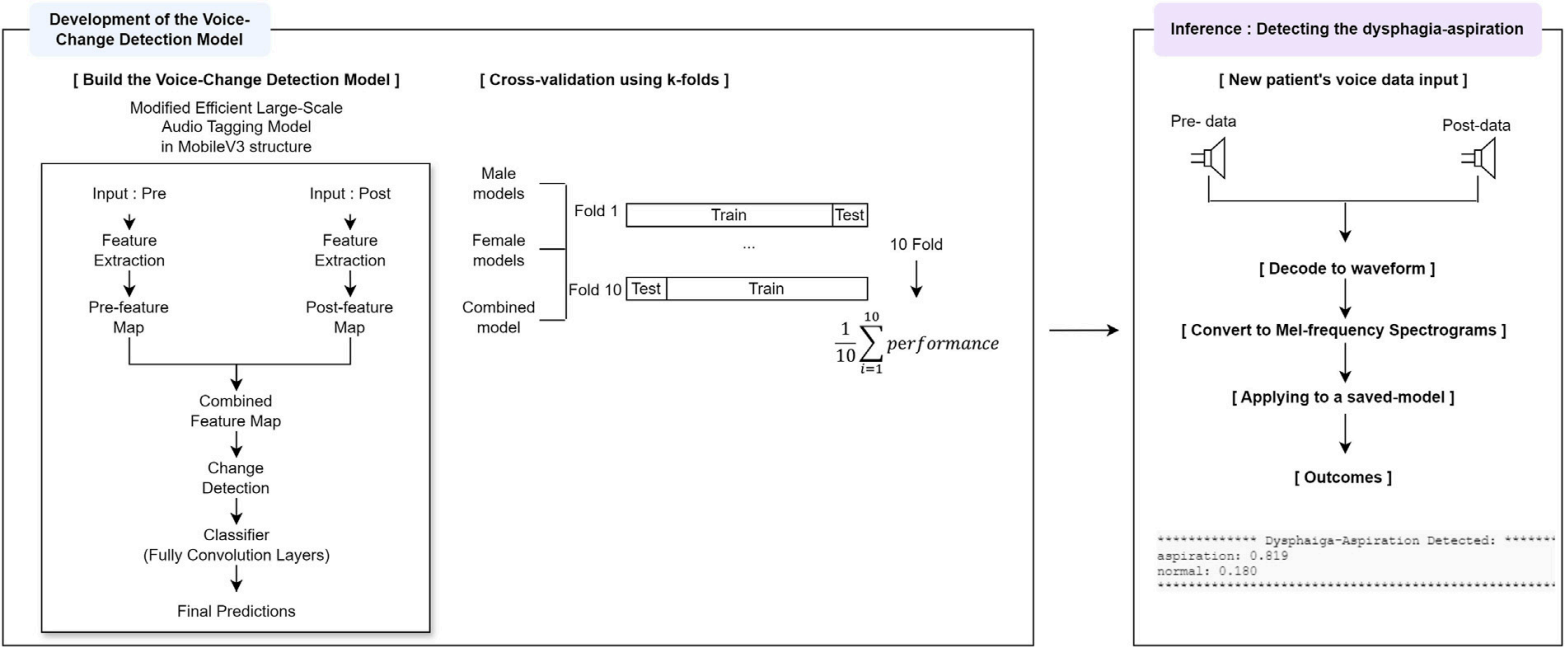
Development of the voice-change detection model and inference windows.

In this study, the trained model used inference to probabilistically determine whether the pre- and post-swallowing audio data of a new patient indicated normal or aspiration. This process involved (i) decoding the mp3 files into waveform; (ii) converting the audio into Mel spectrograms (mels, 128; sample rate, 32,000; window length, 640; hop size, 320); and (iii) loading with trained weights. The model analyzed these spectrograms to predict the likelihood of normal or aspiration risk, outputting the top classification and corresponding probability scores for each audio sample. Figure 3 shows the model structure, performance evaluation and inference architecture of the voice change detection.

### 2.8 Statistical analysis

The characteristics of the study population are presented as mean (SD) for continuous variables and as numbers (%) for nominal variables. Due to non-normality (Shapiro–Wilk test) and sphericity (Mauchly’s test), continuous and nominal variables were analyzed using the Mann–Whitney *U* test and chi-square test, respectively, with the significance set at *p* < 0.05 (Table 1). The primary metric was the area under the receiver operating characteristic (ROC) curve (AUC), supplemented by accuracy, sensitivity, specificity, F1-score, positive predictive value (PPV), negative predictive value (NPV), loss, training accuracy, and training loss, which were reported as means with 95% confidence intervals (CIs) over 10-fold cross-validation (Tables 2, 3). The AUC evaluated the overall model performance under varying thresholds. Accuracy measured the correct predictions among the total samples, as assessed on test and training datasets for accuracy and training accuracy, respectively. Sensitivity was the correct identification rate of actual aspiration cases, whereas specificity was the correct prediction rate of normal cases. The F1-score, calculated as the harmonic mean of precision and sensitivity, was used to evaluate the model’s accuracy. Additionally, the Positive Predictive Value (PPV, same as precision) and Negative Predictive Value (NPV) were calculated, indicating the likelihood of correctly predicted aspiration and correct normal predictions, respectively. AUC, accuracy, sensitivity, and F1-score were calculated using the built-in function features of Python, whereas specificity, PPV, and NPV were derived from the results obtained using a confusion matrix. Loss, which indicated the model error on test data, was computed using binary cross-entropy with logits; training loss was determined similarly, but on the training dataset. Owing to the insufficient amount of aspiration data from female participants, a conservative approach was adopted during performance evaluation by experimentally determining the threshold for normal data to be classified as normal when exceeding 0.5, considering both male and female models as well as the combined model. Statistical analyses and modeling were performed using Python and Google Colaboratory Pro + GPU A100 between May 2023 and March 2024.

**TABLE 1:** Distribution of the study population.

		Normal group		Aspiration group		*p*-value^a^
		N	%	N	%	
**Sex**	Males	41	32.03	52	74.29	<.001^b^ (χ^2^ = 30.76, df = 1)
	Females	87	67.97	18	25.71	
**Age (years)**	Mean ± SD					
	All	61.16 ± 13.00		72.30 ± 12.03		<.001^c^
	Males	63.27 ± 13.57		72.25 ± 11.68		.001^c^
	Females	60.16 ± 12.66		72.44 ± 13.34		.001^c^
Comorbidities						
**Overall (Male + Female)**	Neurological disorders	17 (13.28%)		18 (25.71%)		<.001^d^ (χ^2^ = 36.10, df = 5)
	Gastrointestinal tract and dental disorders	3 (2.34%)		12 (17.14%)		
	Respiratory disorders	4 (3.12%)		9 (12.86%)		
	Other site cancers	7 (5.47%)		3 (4.29%)		
	Aging-associated disorders	12 (9.38%)		8 (11.43%)		
	No medical conditions	85 (66.41%)		20 (28.57%)		
**Male**	Neurological disorders	5 (12.20%)		11 (21.15%)		.002^d^ (χ^2^ = 18.54, df = 5)
	Gastrointestinal tract and dental disorders	1 (2.44%)		12 (23.08%)		
	Respiratory disorders	2 (4.88%)		8 (15.38%)		
	Other site cancers	2 (4.88%)		2 (3.85%)		
	Aging-associated disorders	5 (12.20%)		6 (11.54%)		
	No medical conditions	26 (63.41%)		13 (25.00%)		
**Female**	Neurological disorders	12 (13.79%)		7 (38.89%)		.140^d^ (χ^2^ = 8.31, df = 5)
	Gastrointestinal tract and dental disorders	2 (2.30%)		0 (0.00%)		
	Respiratory disorders	2 (2.30%)		1 (5.56%)		
	Other site cancers	5 (5.75%)		1 (5.56%)		
	Aging-associated disorders	7 (8.05%)		2 (11.11%)		
	No medical conditions	59 (67.82%)		7 (38.89%)		

^a^ Sex and comorbidities were analyzed using the chi-squared test, while age was analyzed using the Mann-Whitney *U* test.

^b^ To eliminate sex bias, male-only and female-only models were constructed.

^c^ Dysphagia is a condition commonly found in the elderly, and efforts were made to minimize age bias. Nevertheless, participants under the age of 40 were not included in order to remove as much age bias as possible, despite the remaining age distribution difference between the control and aspiration groups.

^d^ The presented comorbidities show possible diseases that can come with dysphagia, revealing clear differences in disease characteristics between the normal and aspiration groups. This table is added to clearly and briefly show these comorbidity profiles for analysis.

**TABLE 2:** Performance of male and female models.

Sex	Learning rate	Batch size	Results	AUC^a^	Accuracy	Sensitivity	Specificity	F1 score	PPV^a^	NPV^a^	Loss^b^	Train accuracy^c^	Train loss^b^ ^,^ ^c^
**Male**	**5.00e-05**	**32**	**Mean**	0.8117	84.72	91.40	70.95	0.8509	80.46	89.59	0.4042	100.00	0.2005
			**(95% CI)**	(0.7520, 0.8715)	(80.27, 89.18)	(82.61, 100.19)	(58.78, 83.12)	(0.7771, 0.9247)	(72.74, 88.19)	(82.94, 96.25)	(0.3583, 0.4500)	(100.00, 100.00)	(0.1979, 0.2030)
			**Max**	0.9654	96.30	100.00	95.71	0.9487	92.50	100.00	0.5386	100.00	0.2066
		**16**	**Mean**	0.8238	85.66	92.42	72.35	0.8612	81.37	91.22	0.3724	100.00	0.1378
			**(95% CI)**	(0.7761, 0.8716)	(81.86, 89.45)	(85.63, 99.20)	(61.37, 83.33)	(0.8041, 0.9184)	(74.50, 88.25)	(85.07, 97.37)	(0.3172, 0.4275)	(100.00, 100.00)	(0.1346, 0.1409)
			**Max**	0.9224	93.06	100.00	95.00	0.9484	91.15	100.00	0.5356	100.00	0.1448
	**3.00e-05**	**32**	**Mean**	0.8274	85.14	90.90	74.59	0.8519	80.93	90.59	0.4356	100.00	0.2433
			**(95% CI)**	(0.7576, 0.8973)	(79.05, 91.24)	(80.73, 101.06)	(62.00, 87.18)	(0.7673, 0.9365)	(72.26, 89.61)	(82.94, 98.25)	(0.3822, 0.4889)	(100.00, 100.00)	(0.2415, 0.2451)
			**Max**	0.9491	93.98	100.00	91.79	0.9467	93.02	100.00	0.5967	100.00	0.2468
		**16**	**Mean**	0.8319	85.67	90.46	75.92	0.8533	81.12	89.97	0.3931	94.00	0.1954
			**(95% CI)**	(0.7828, 0.8811)	(81.25, 90.09)	(82.95, 97.96)	(66.49, 85.36)	(0.7796, 0.9270)	(72.87, 89.38)	(84.52, 95.42)	(0.3397, 0.4465)	(84.22, 103.78)	(0.1924, 0.1983)
			**Max**	0.9317	95.36	100.00	91.79	0.9673	94.71	100.00	0.5453	100.00	0.2029
**Female**	**5.00e-05**	**32**	**Mean**	0.7331	83.79	56.09	90.53	0.4972	56.48	88.80	0.4016	96.67	0.1299
			**(95% CI)**	(0.6244, 0.8418)	(75.62, 91.95)	(35.37, 76.81)	(85.24, 95.81)	(0.3102, 0.6843)	(32.25, 80.71)	(79.47, 98.13)	(0.2821, 0.5211)	(90.13, 103.20)	(0.1268, 0.1331)
			**Max**	0.9688	99.43	100.00	100.00	0.9677	100.00	100.00	0.7487	100.00	0.1421
		**16**	**Mean**	0.7128	83.26	51.31	91.25	0.4584	50.07	87.92	0.4215	95.00	0.0612
			**(95% CI)**	(0.6053, 0.8204)	(74.81, 91.70)	(30.81, 71.81)	(86.71, 95.79)	(0.2930, 0.6237)	(29.91, 70.23)	(78.17, 97.67)	(0.2464, 0.5967)	(85.20, 104.80)	(0.0584, 0.0640)
			**Max**	0.9375	98.86	100.00	100.00	0.9333	100.00	100.00	0.9755	100.00	0.0719
	**3.00e-05**	**32**	**Mean**	0.7294	83.77	56.53	89.34	0.4607	44.36	89.77	0.4165	100.00	0.1856
			**(95% CI)**	(0.6164, 0.8424)	(76.35, 91.20)	(33.88, 79.19)	(84.17, 94.51)	(0.2740, 0.6473)	(26.56, 62.15)	(81.50, 98.03)	(0.3065, 0.5265)	(100.00, 100.00)	(0.1828, 0.1884)
			**Max**	0.9188	95.45	87.50	96.51	0.7778	84.38	98.72	0.7717	100.00	0.1965
		**16**	**Mean**	0.6975	82.00	47.99	91.51	0.3860	44.49	87.21	0.4316	100.00	0.1172
			**(95% CI)**	(0.5844, 0.8105)	(72.88, 91.13)	(23.71, 72.27)	(87.95, 95.06)	(0.2389, 0.5331)	(30.39, 58.60)	(75.79, 98.64)	(0.2708, 0.5925)	(100.00, 100.00)	(0.1139, 0.1204)
			**Max**	0.9192	93.92	100.00	97.80	0.7179	74.39	100.00	1.0260	100.00	0.1301

^a^ AUC (Area Under the ROC Curve), PPV (Positive Predictive Value), NPV (Negative Predictive Value).

^b^ Loss function: Binary cross entropy with logits loss function.

^c^ Except for ‘Train Accuracy’ and ‘Train Loss’, all the parameters represent the test datasets for the 10-fold cross-validation. Additionally, all metrics, excluding AUC, F1 score, Loss, and Train Loss, were calculated and expressed as percentages.

**TABLE 3:** Combined model (male + female).

Learning rate	Batch size	Results	AUC	Accuracy	Sensitivity	Specificity	F1 score	PPV	NPV	Loss	Train accuracy	Train loss
**5.00e-05**	**32**	**Mean**	0.7874	81.31	72.65	84.82	0.7235	74.59	85.37	0.4550	95.00	0.0819
		**(95% CI)**	(0.7233, 0.8514)	(74.94, 87.69)	(60.90, 84.40)	(77.08, 92.56)	(0.6322, 0.8148)	(63.94, 85.24)	(79.59, 91.14)	(0.3442, 0.5657)	(85.20, 104.80)	(0.0797, 0.0841)
		**Max**	0.8920	91.82	91.89	97.73	0.8819	94.49	96.42	0.7721	100.00	0.0889
	**16**	**Mean**	0.7746	81.25	67.87	87.06	0.7006	76.56	84.40	0.5323	72.50	0.0285
		**(95% CI)**	(0.7046, 0.8447)	(74.51, 87.98)	(53.58, 82.16)	(80.70, 93.42)	(0.5836, 0.8176)	(64.48, 88.65)	(78.38, 90.43)	(0.3726, 0.6919)	(48.89, 96.11)	(0.0269, 0.0302)
		**Max**	0.8895	92.45	92.69	99.68	0.9097	98.37	92.07	1.0099	100.00	0.0348
**3.00e-05**	**32**	**Mean**	0.7971	81.61	76.51	82.92	0.7401	72.90	86.84	0.4309	100.00	0.1404
		**(95% CI)**	(0.7303, 0.8640)	(74.60, 88.62)	(66.93, 86.08)	(74.81, 91.02)	(0.6505, 0.8298)	(62.16, 83.64)	(81.40, 92.29)	(0.3553, 0.5064)	(100.00, 100.00)	(0.1382, 0.1425)
		**Max**	0.9360	92.41	98.40	95.97	0.9069	88.99	98.92	0.6822	100.00	0.1479
	**16**	**Mean**	0.7997	82.64	73.26	86.67	0.7486	78.27	85.24	0.4328	85.00	0.0715
		**(95% CI)**	(0.7450, 0.8543)	(76.57, 88.72)	(65.19, 81.34)	(79.61, 93.72)	(0.6731, 0.8241)	(67.40, 89.13)	(80.09, 90.38)	(0.3369, 0.5287)	(64.08, 105.92)	(0.0691, 0.0740)
		**Max**	0.8982	94.51	84.62	99.36	0.8889	95.77	94.46	0.7302	100.00	0.0795

^a^ This model also used the loss function (Binary cross-entropy with logits) and all parameters, except ‘Train Accuracy’ and ‘Train Loss,’ refer to the test datasets in the 10-fold cross-validation.

## 3 Results

### 3.1 Study population distribution

Table 1 shows the distribution of the study population in each group.

### 3.2 Model performance

Separate models were constructed for each sex to address individual and sex-related biases, as shown in Table 2. For males, the average AUC values ranged from 0.8117 to 0.8319, peaking at a learning rate of 3.00e-05 and a batch size of 16. For females, average AUC values ranged from 0.6975 to 0.7331, optimized at a learning rate of 5.00e-05 and batch size of 32.

**FIGURE 4:**
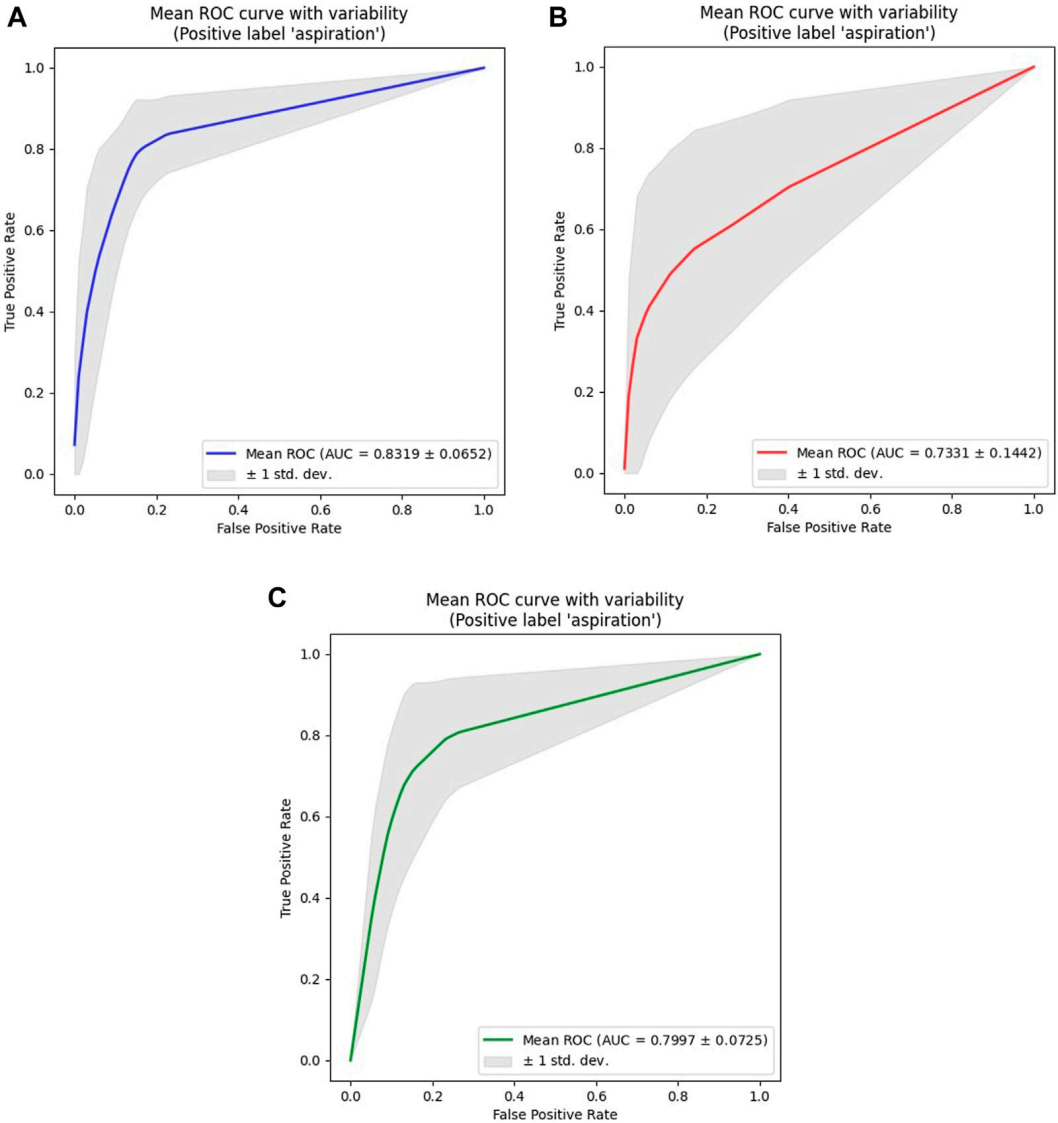
ROC analysis results for all models. **(A)** Male Models. **(B)** Female Models. **(C)** Combined (Male + Female) Models. The ROC curve shown represents the average ROC curve for the 10 folds under the parameter combination (learning rate, batch size) that resulted in the highest AUC value for models according to sex (male, female, combined). For the male model, the highest AUC value was 0.8319, achieved with a learning rate of 3.00e-5 and a batch size of 16. For the female model, the highest AUC value was 0.7331, achieved with a learning rate of 5.00e-5 and a batch size of 32. For the combined model, the highest AUC value was 0.7997, achieved with a learning rate of 3.00e-5 and a batch size of 16. Among the sex-specific models, the male model showed the highest overall AUC value, as it had the most even distribution of data across groups. Although the female model displayed an accuracy similar to that of the male model, a significant imbalance between the normal group and the aspiration group led to the relatively lowest AUC value.

Given the limitations in building a machine-learning model for aspiration detection in females owing to the small number of female participants with aspiration, a combined model was created by maintaining sex proportions in each group (Table 3). The ROC curves showed the parameter combinations of the learning rate and batch size that achieved the highest performance for each of the models according to sex (male, female, and combined) at the 150th epoch (Figure 4).

## 4 Discussion

This study aimed to develop a non-invasive, deep learning-based detection system that allows for the periodic monitoring of swallowing conditions in patients with dysphagia during their daily activities. In our study, we modified the well-known EfficientAT (Schmid et al., 2023a; 2023b), which is renowned for solving sound classification problems, to create a new model capable of detecting voice changes. When applied to voice changes pre- and post-swallowing in patients with dysphagia-aspiration at our hospital, the model attained an average AUC value of >0.80 for males, with the best performing model achieving an AUC of 0.8319. However, for females, the average AUC value was approximately 0.70, with the highest AUC value from the optimal parameter combination being 0.7331 only. This lower performance could be attributed to the limited number of female participants in the aspiration group; nevertheless, the combined model designed to encompass both male and female voices consistently achieved AUC values exceeding 0.75, with the highest value reaching 0.7997. The difference in performance between the male and female models is primarily due to the limited number of female patients with aspiration, totaling only 18, and the severe data imbalance between the normal and aspiration groups. This imbalance implies that even with oversampling, there were limitations to adequate learning about aspiration. Therefore, the lower AUC performance in females can be attributed to the limitations of sample sampling and the characteristics of the voice data.

The process of swallowing involves several phases, and disruptions during the pharyngeal phase can lead to serious complications, such as aspiration pneumonia, due to food entering the airway (Lundy et al., 1999; Matsuo and Palmer, 2008; Sasegbon and Hamdy, 2017). Protective reflexes, such as the tilting of the epiglottis and closure of the vocal cords, are crucial in preventing potentially life-threatening aspiration (Shaker et al., 1990; Shaker, 1995; Van Daele et al., 2005; Matsuo and Palmer, 2008). Reflecting these mechanisms, the PAS is commonly used as a diagnostic criterion for dysphagia. It classifies the extent of penetration and aspiration based on how the bolus interacts with the vocal cords and enters the airways (Rosenbek et al., 1996). In addition, a study reported a significant positive correlation between the degree of airway penetration and the occurrence of airway protective responses such as coughing and throat clearing (aspiration amounts (%) - odds ratio: 1.12, 95% CI: 1.09–1.16, *p* < 0.001) (Curtis et al., 2023). In a study examining the relationship between swallowing and respiratory and phonatory functions, individuals with airway penetration showed significantly reduced laryngeal elevation, shorter Maximum Phonation Time (MPT), and lower chest expansion scores at the 10th rib level, indicating compromised respiration. These findings suggest a clear link between swallowing difficulties and respiratory and vocal function impairment (Yamaguchi et al., 2018). Based on this, the study hypothesized that aspiration after swallowing meals leads to changes in the airway and respiration, affecting the vocal cords. Consequently, we predicted that there would be more noticeable changes in the vocal characteristics pre- and post-swallowing meals in the aspiration group than in healthy individuals.

From this perspective, voice analysis programs have been explored to identify vocal characteristics as biomarkers for dysphagia monitoring (Ryu et al., 2004; Waito et al., 2011; Santos et al., 2015; Kang et al., 2018; Dos Santos et al., 2022; Park et al., 2022; Song et al., 2022). Studies have found significant changes in vocal parameters like Relative Average Perturbation (RAP) Jitter, Shimmer Percentage (SHIM), Noise-to-Harmonics Ratio (NHR), and Voice Turbulence Index (VTI) in individuals at high risk for aspiration, with these parameters showing high sensitivity in predicting aspiration risk (Ryu et al., 2004; Song et al., 2022). A notable study using ANOVA found a significant interaction in RAP values pre- and post-swallowing between groups at risk and not at risk for aspiration (Kang et al., 2018). Furthermore, correlations between various scales like Grade, Roughness, Breathiness, Asthenia, Strain scale (GRBAS scale), PAS, Videofluoroscopic Dysphagia Scale (VDS), American Speech Language Hearing Association-National Outcome Measurement System (ASHA-NOMS), and vocal parameters have been reported (Waito et al., 2011; Song et al., 2022). Advanced analytical techniques like logistic regression, decision trees, random forests, and support vector machines, particularly XGBoost using vocal parameters, showed the best performance (Park et al., 2022). However, these studies have practical limitations in clinical settings because of the need to manually extract numerical data from the vocal parameters for analysis.

To overcome these limitations and develop programs that are more suitable for clinical environments or medical devices, attempts have been made to convert patients’ voices into Mel spectrograms and train deep learning models to minimize manual work and analyze the voice signals themselves (Kim et al., 2023; Kim et al., 2024). One study focused on creating a dysphagia predictive model using four vocalizations: prolonged vowel phonation, voluntary cough, pitch elevation, and counting (Kim et al., 2023). Based on previous research on post-swallowing voice indicators, our research team constructed a machine-learning model using the EfficientAT model to detect the occurrence of aspiration based on the post-swallowing voices of patients. The model with the best performance was “mn30_as,” built using transfer learning from a pre-trained model and evaluated using 10-fold cross-validation. Based on the AUC metric, the results showed the following performances: 0.8010 (95% CI: 0.6598–0.9432; max: 1.0000) for the male model, 0.7572 (95% CI: 0.6578–0.8567; max: 0.9779) for the female model, and 0.8361 (95% CI: 0.7667–0.9056; max: 0.9541) for the combined male and female model. With respect to accuracy, the male, female, and combined models averaged 85.13% (95% CI: 78.07–92.19; max: 100.00), 69.16% (95% CI: 61.76–76.57; max: 88.00), and 77.98% (95% CI: 70.07–85.89; max: 92.45), respectively. (Schmid et al., 2023a; 2023b; Kim et al., 2024). Overall, the pre- and post-swallowing voice change detection model presented in this paper exhibited higher accuracy performance. However, in terms of the AUC, although an improvement was observed in the male model, it deteriorated in the female model. The factors contributing to these differences are as follows. First, cutting both pre- and post-data into 2-s segments and combining them, which amplified the data count, resulted in male data achieving a similar or better balance between the normal and aspiration groups compared to the post-model, thus showing higher or similar AUC values. In contrast, the gap in the amount of female data between the normal and aspiration groups widened, leading to higher accuracy but lower AUC values. Second, the process of cutting into 2-s segments served to filter out noise present across the entire voice recording, especially normal data. Third, Mel spectrogram analysis revealed clear vocal pattern distinctions between the normal and aspiration groups in females, although the swallowing effects were subtle. Conversely, males showed stark voice data contrasts between the groups, with pronounced swallowing effects. These factors likely affected performance; however, limited data on female patients with aspiration prevent accurate representation. Fourth, the model size may also have influenced the performance; for the pre- and post-swallowing voice change detection model, only version 2.0, which could be used owing to CUDA memory issues, whereas for the post model, version 3.0 was feasible. Although there were factors affecting performance compared to previous studies, we sought to improve the model by considering the following points. Age-related lung capacity limitations among dysphagia patients often resulted in incomplete 5-s recordings, affecting data uniformity. (Bowdish, 2019; Tong and Sataloff, 2022). Standardizing segment length to 2 s improved analysis consistency. Lastly, dysphagia is influenced significantly by changes such as aspiration after swallowing, highlighting its importance in the pathophysiology of this condition. The post-swallowing model has limitations in adequately reflecting these pathophysiological aspects of the disease. (Matsuo and Palmer, 2008; Saitoh et al., 2018).

Therefore, the significance of this study lies in its ability to detect the differences in voice changes pre- and post-swallowing. To achieve this, a combination of voice data from patients pre- and post-swallowing was used to extract voice features and identify variations. This suggests that changes in voice characteristics pre- and post-swallowing can serve as significant indicators for detecting dysphagia-aspiration, in addition to factors such as underlying muscle weakness in the throat and aspiration due to saliva, which are common among patients with dysphagia (Jang et al., 2013; Sasegbon and Hamdy, 2017; Yamaguchi et al., 2019). To enable integration into mobile or medical devices, pre- and post-audio data, patient-anonymized identifiers, and normal or aspiration status were organized hierarchically and stored in a compact file format known as HDF5. This approach aims to lighten the data (The-HDF-Group, 2006; Ji et al., 2020). Additionally, to minimize the size and capacity of the model while maximizing the efficiency for implementation in mobile and medical device environments, the MobileNetV3 model was utilized (Howard et al., 2019). Furthermore, to minimize information loss within voice files and to standardize the audio data and lightweight format for medical devices, all voice files were converted and saved in mp3 format at 64 kbps with two mono-channel configurations (Pollak and Behunek, 2011; Fuchs and Maxwell, 2016; Sun, 2021). While it is known that compressing audio files to MP3 can result in a significant loss of quality, previous research has shown that when the compression rate for f0 measurements is between 56 and 320 kbps, the average error is less than 2%, with median errors as low as 0.5%. In addition, errors in measuring the pitch range and level have been reported to be below 1%. Therefore, considering these findings, along with the medical device environment and compression rates, we standardized the use to 64 kbps (Fuchs and Maxwell, 2016).

In addition to studies that utilize the patients’ voice for diagnosing and monitoring dysphagia, attempts have been made to use various non-invasive methods (Daniels et al., 1997; Mari et al., 1997; Nishiwaki et al., 2005; Groher et al., 2006; Clave et al., 2008; Rofes et al., 2012; Somasundaram et al., 2014; Brodsky et al., 2016; Festic et al., 2016; Zuniga et al., 2018; Riera et al., 2021). In studies where patients consumed boluses of varying viscosities (ranging from 5 to 20 mL) at the bedside, the sensitivity ranged from 88.2% to 100.0%, and the specificity ranged from 28.8% to 81.39% for aspiration (Clave et al., 2008; Rofes et al., 2012; Riera et al., 2021). Other methods such as the assessment of dysphonia, dysarthria, gag reflex, volitional cough, and voice changes after swallowing demonstrated a sensitivity of 30.8%–92.3% and a specificity of 60.6%–87.9% (Daniels et al., 1997). The Eating Assessment Tool-10 (EAT-10) showed a sensitivity of 77.8% and specificity of 73.1% (Zuniga et al., 2018). In a meta-analysis of the Bedside Water Swallow Test, the overall sensitivity for airway response or voice change in relation to volume was reported to be 63% or higher (Brodsky et al., 2016). Additionally, various studies have reported the correlation between indicators of aspiration and dysphagia using methods and measures such as the 3-oz water swallow test (Mari et al., 1997; Nishiwaki et al., 2005; Groher et al., 2006; Somasundaram et al., 2014; Festic et al., 2016). These methods require specialized knowledge to monitor the patients’ swallowing status in daily life, making it difficult to apply them in practical settings. Furthermore, the study results indicate that these findings are comparable to those obtained using non-invasive diagnostic methods.

In contrast to the aforementioned studies utilizing various non-invasive methods for diagnosing dysphagia, which often require expert judgment and controlled examination settings, our research uniquely employs only the patients’ voice, specifically the simple vocalization ‘ah∼‘. (Daniels et al., 1997; Mari et al., 1997; Nishiwaki et al., 2005; Groher et al., 2006; Clave et al., 2008; Rofes et al., 2012; Somasundaram et al., 2014; Brodsky et al., 2016; Festic et al., 2016; Zuniga et al., 2018; Riera et al., 2021). This approach enables broad applicability across different populations, leveraging global considerations and disease characteristics. Our model processes voice recordings within approximately 2–5 s, providing a rapid and straightforward assessment of swallowing function, as demonstrated in our inference implementation. Above all, dysphagia is a condition occurring during the intake of food and liquids in daily life, where its management is crucial not only for the condition itself but also to minimize aspiration events and ensure adequate nutrition intake. (Matsuo and Palmer, 2008; Saitoh et al., 2018). In this sense, the state of dysphagia necessitates continuous monitoring of changes in the patient’s daily life and immediate intervention based on its status. Our research model is significant as it can assess dysphagia status in daily life without specialist intervention. We plan to anonymize and manage these diagnostic results in a database accessible to clinical practitioners with patient consent, offering a supplementary means to diagnose dysphagia-aspiration within routine clinical parameters.

Also, in this study, we used the PAS scale from VFSS examinations as the gold standard to distinguish between normal and aspiration cases. (Lind, 2003; Saitoh et al., 2018). Therefore, integrating our aspiration detection algorithm based on patient voice with the PAS scale allows comprehensive assessment of daily pharyngeal conditions. (Rosenbek et al., 1996). This integrated approach enhances diagnostic accuracy by combining voice-based assessments with visual examination results. Additionally, linking with tools like the Functional Oral Intake Scale (FOIS) for assessing oral intake function and the Mini Nutrition Assessment (MNA) for evaluating nutritional deficiencies will provide insights into dysphagia’s impact on nutrition. (Nordio et al., 2020; Ueshima et al., 2021). This comprehensive approach establishes a foundation for personalized interventions tailored to individual patient conditions in clinical settings.

This deep learning-based study was developed to detect aspiration during swallowing by detecting changes in voice pre- and post-swallowing. The expected effects of this research are as follows: First, patients will be able to monitor their daily swallowing status and receive personalized guidance for dysphagia-related meals and rehabilitation training through this detection algorithm. Second, clinical doctors will be able to monitor daily data on changing dysphagia status in patients’ dietary habits, enabling a more accurate diagnosis and reducing the time required for diagnosis. Third, it is expected to lay the foundation for the development of an integrated rehabilitation training system that combines diagnosis, treatment, monitoring, and management through integration with mobile platforms and medical devices.

## 5 Limitations

This study had several limitations. First, owing to the limited number of patients, we were unable to create a separate validation set and therefore employed 10-fold cross-validation at a 9:1 train-test ratio. Second, the small sample size of 18 female patients with aspiration resulted in lower performance compared to the male and combined models. Additionally, the data for women in the aspiration group were limited to 18 individuals, making it difficult to consider this as representative of dysphagia. Data imbalance between the normal and aspiration groups was also an issue. Third, the process of recording voices in hospitals is not included in standard examination procedures, and there are limitations to data collection because most patients with dysphagia are older. In particular, collecting data from healthy individuals poses challenges owing to difficulties in visiting hospitals, and a process to extend and collect data from the general population is necessary, considering the future commercialization of medical devices. There were limitations to this process because of the lack of uniformity in the type of food consumed by the research participants and the recording devices used. To overcome these limitations, all collected data were standardized to the mp3 format at 64 kbps for analysis, and a high similarity (>0.85) was observed in the homogeneity evaluation calculated by cosine similarity according to each device. Fourth, we adjusted various threshold values to determine if samples from the current study population were normal or aspiration. We identified a threshold where our model consistently showed high performance across all metrics, especially AUC, accuracy, sensitivity, and specificity. Specifically, we set the criterion that voice samples with a probability of normal exceeding 0.5 are classified as normal; otherwise, they are classified as aspiration. However, these thresholds are based on potentially insufficient data. Future plans include expanding the study across multiple institutions and using a larger dataset of patients with dysphagia. This expansion aims to not only enhance the model performance, establish comprehensive datasets, and validate the generalization performance in various settings but also to refine and optimize threshold settings. Ultimately, these efforts aim to improve the reliability of medical and mobile applications, providing a more robust foundation for clinical use.

## 6 Conclusion

This study suggests the possibility of developing a supplemental program that can detect pre- and post-swallowing changes and monitor dysphagia status using an uncomplicated voice analysis in patients with dysphagia. Through a real-time monitoring system for patients with dysphagia using this analysis, clinicians and experts in the field can be provided with the parameters of patients’ daily indicators of their conditions, enabling appropriate interventions tailored to the patients’ current state. This innovative approach may minimize patient burden, maximize treatment effectiveness, and overcome language barriers by utilizing simple vocalizations such as “ah∼” for monitoring swallowing status. Monitoring the aspiration status in the daily life of patients with dysphagia is expected to improve their quality of life, reduce the incidence of secondary diseases caused by dysphagia, and enhance the treatment effectiveness for comorbidities.

## Data Availability

The raw data supporting the conclusions of this article will be made available by the authors, without undue reservation.

## Abbreviations

AUC, Area Under the ROC Curve; VFSS, Videofluoroscopic Swallowing Study; PAS, Penetration-Aspiration Scale; STROBE, Strengthening the Reporting of Observational Studies in Epidemiology; YP, Yoplait yogurt; SF, Small Fluid; SBD, Semi-Blended Diet; FT3, Fluid Thickening with level 3; LF, Liquid Food; HDF5, Hierarchical Data Format 5; Efficient AT model, Efficient Pre-trained CNNs for Audio Pattern Recognition; PPV, Positive Predictive Value; NPV, Negative Predictive Value; CIs, Confidence Intervals; MPT, Maximum Phonation Time; RAP, Relative Average Perturbation; SHIM, Shimmer Percentage; NHR, Noise-to-Harmonics Ratio; VTI, Voice Turbulence Index; GRBAS, Grade, Roughness, Breathiness, Asthenia, Strain scale; VDS, Videofluoroscopic Dysphagia Scale; ASHA-NOMS, American Speech language Hearing Association-National Outcome Measurement System; EAT-10, Eating Assessment Tool-10; FOIS, Functional Oral Intake Scale; MNA, Mini Nutrition Assessment.
